# Stakeholders’ perspectives on competency-based education program in Africa: A qualitative study

**DOI:** 10.4102/hsag.v29i0.2416

**Published:** 2024-11-18

**Authors:** Barbara M. Dube, Ntombifikile G. Mtshali

**Affiliations:** 1 School of Nursing and Public Health, College of Health Sciences, University of KwaZulu-Natal, Durban, South Africa

**Keywords:** competency-based education, nursing education, teaching and learning, nursing student, primary health care, curriculum, qualitative study

## Abstract

**Background:**

Competency-based education (CBE) is adopted to reform health professionals’ education. The goal is to produce graduates who are not only competent in their fields but also equipped with the necessary social accountability skills for safe practice.

**Aim:**

The study aimed to explore nursing education stakeholders’ perspectives on competency-based primary healthcare (PHC)-oriented nursing education programmes in the South African context.

**Setting:**

The setting for this study was a higher education institution in South Africa.

**Methods:**

A descriptive qualitative method was used in this study. After taking ethical clearance from the university ethics committee, data were purposively collected and theoretically sampled from 40 participants through focus group discussion, individual interviews and document analysis.

**Results:**

The data findings reported categories that emerged from this study, which include the attributes of a competency-based PHC-oriented programme and the outcomes of a competency-based PHC-oriented programme.

**Conclusion:**

In the context of this study, the concept of CBE was portrayed as a programme with strong social accountability that aims at addressing priority health needs surrounding communities and ensuring that the health needs of the community are met while providing health delivery service to communities in their natural environment.

**Contribution:**

These findings add to the growing evidence base around CBE as an approach to strengthen PHC-oriented health services in low- and middle-income countries, a key aspect of which is providing service within communities. This enables the accessibility of quality healthcare closer to where people live and work to achieve Sustainable Development Goal number three.

## Introduction

### Background

The adoption of competency-based education (CBE) is one of the recommendations in the Lancet report to reform health professionals’ education, including nursing education, to strengthen health systems (Bvumbwe & Mtshali 2018; Frenk et al. 2010). Competency-based education resulted from a growing demand for public accountability and transparency in curricula (Zeller et al. 2016) and equipped with a new set of competencies that translate theory into practice, especially among newly recruited nurses (Chen, Zhang & Li 2022; Wyne, Zhang & Farahani 2021). Furthermore, implementing CBE assists in the justification for training in increasingly costly education, as central to education are the needs of the population and health system (Zeller et al. 2016). Competency-based curriculum (CBC) addresses the concern about linking theory to practice to improve the quality of education and graduates produced (Gervais 2016; Wyne et al. 2021). Nursing education institutions, as socially accountable institutions, have an obligation to align their education programmes to the priority health needs and concerns of the surrounding community, region and nation (Rispel 2016). According to Boelen (2016), socially accountable institutions prioritise education, research and service activities that respond to the current and future health needs and challenges of the population served and the health care system’s demands.

In South Africa, primary health care (PHC) re-engineering is one of the priorities of the health care system to address the inequities of the past, widen access to health care for universal health coverage and address the burden of disease (Saloojee 2020). Primary health care re-engineering refers to the process of restructuring and enhancing the delivery of primary health care services to meet a population’s health needs better. This approach aims to improve the accessibility, quality, efficiency and effectiveness of essential health services, particularly at the community level (World Health Organization 2011). It involves a range of strategies and interventions that focus on strengthening various aspects of PHC. Rispel (2015) states that PHC remains a cornerstone of health reforms for universal health coverage and improved population health outcomes. Therefore, the nursing education institutions, as producers of the bulk of the health workforce in South Africa, have to align their education programmes to the philosophy underpinning the health care system and ensure that graduates produced are equipped with skills essential to respond to national health priorities and population health needs (Frenk et al. 2010; Muraraneza, Mtshali & Mukamana 2017). Hence, there is growing interest in adopting competency-based nursing education. According to Tan et al. (2018), the nursing profession has no option but to adopt CBE to produce graduates of high quality who are equipped with elevated levels of competencies to drive the agenda of the health care system. The concept of CBE is defined differently and implemented in a consistent manner (Muraraneza & Mtshali 2018a). It is sometimes used interchangeably with outcomes-based, mastery-based, proficiency-based and performance-based education (Gravina 2017). In this article, CBE is defined as an outcomes-based, context-informed and student-centred approach to curriculum design, teaching and learning (Schumacher & Risco 2017; Zeller et al. 2016).

Literature reflects different perspectives on CBE, both positive and negative. The negative aspect of CBE reported is that it is not helpful in teaching interpersonal and vocational skills (Van Griethuijsen et al. 2020). Competency-based education brings new hope of narrowing the gap between theory and practice, taking into consideration the gap in the traditional content-driven curriculum between what nurses learn, know and do and the expectations of the patients, workplace stakeholders and licensing bodies (Barton, Bruce & Schreiber 2018; Tan et al. 2018). The curriculum is informed by the competencies required from the graduates, and teaching, learning and assessment directly focus on the acquisition of expected graduate competencies. It emphasises what the students should know, understand and demonstrate to be certified competent. Furthermore, it prepares the students on how to adapt to life beyond formal education (Tan et al. 2018), as it equips the graduates with discipline-specific sets of skills and transferable life skills essential for survival in the ever-changing world of work (Muraraneza & Mtshali 2018b). Competency-based education is a performance-based approach to reform nursing education to respond to the health system and the population’s health needs (Tan et al. 2018). The primary focus in CBE is the mastery of a set of competencies presented in the form of measurable behaviours, knowledge, actions and skills essential to the nursing practice (Caccia et al. 2015b; Kiguli et al. 2014; Muraraneza, Mtshali & Mukamana 2018). Competency-based education is considered a need-driven curriculum. It emerged that, with CBC, students take a more active role in their learning, thereby reducing redundancies.

Competency-based education is student-centred learning (Caccia, Nakajima & Kent 2015a; Manske 2021), where learning is personalised with students taking a significant role in teaching and learning. Seemingly, students produced in such a programme are skilled with transferable life skills such as decision-making, critical thinking and problem-solving skills. Nurses produced in a competency-based programme could contribute positively to the health care system, which is challenged by a lack of quality nurses. Although CBC has been offered for several years, students have reported a lack of proper guidance and support in the clinical environment. It emerged that students have often found themselves having to rely on the skeleton staff for clinical supervision and guidance because of staff shortages and heavy workloads (Eta et al. 2011; Katowa-Mukwato et al. 2014). Furthermore, several researchers believe that, for the successful implementation of CBC, there should be adequate resources for teaching and learning, including skilled educators (Katoue & Schwinghammer 2020; Stoffman 2022). Literature, however, reports a lack of a standard definition of CBE (Barton et al. 2018; Woeber 2018). There is also a lack of empirical research on the effectiveness of this outcomes-based approach in students’ acquisition of desirable competencies because of a lack of conformity around standards and theoretical backing (Gervais 2016).

In South Africa, there is a paucity of studies on the perspectives of faculties and students about competency-based PHC nursing education. Therefore, the study aimed to explore nursing education stakeholders’ perspectives on competency-based PHC-oriented nursing education programmes in the South African context.

## Research question

*How do nursing education stakeholders understand competency-based PHC-oriented nursing education programmes in the South African context?*

## Research methods and design

### Study design and setting

Underpinned by the constructivist paradigm, the study adopted a qualitative research design to gain an in-depth insight into the conceptualisation and outcomes of implementing a competency-based PHC-oriented curriculum. This design is strategic in nature as it considers the methods of exploring an area of human experience to understand humans’ world perception (Roberts & Priest 2010). The study was conducted in a university-based nursing and midwifery department in a selected higher educational institution in KwaZulu-Natal. This Nursing and Midwifery Department has a student population of 315 registered in both undergraduate and postgraduate programmes at various levels. It has shifted from a teacher-centred to a student-centred curriculum by combining problem-based and community-based approaches. These approaches aim to produce competent and prepared graduates for clinical practice.

### Study population and sample

The study population consisted of 315 students and 20 faculty members. Purposive sampling was used to select those students who are conversant with CBE and who will be able to provide the information the researchers are looking for. All the educators, programme coordinators and a curriculum expert were invited to participate in the study. The study sample constituted 8 nurse educators, 1 curriculum expert, 1 programme coordinator, 2 level coordinators, 2 clinical facilitators, 2 preceptors and 24 undergraduate nursing students registered in a bachelor’s degree programme. The total sample size was 40 (*n* = 40). The inclusion criteria for students and educators were all undergraduate nursing and midwifery students and educators in the study setting. In addition, the student must have been at least 6 months in the programme with clinical exposure and exposure to CBE. The exclusion criteria were all those undergraduate nursing and midwifery students registered as students with less than 6 months in the programme and with no clinical experience. The participants were purposively selected based on their ability to provide the information being sought and later theoretically sampled as determined by their involvement in and experiences of CBC. Eight students were purposively selected from each level of study (levels two, three and four), while all the educators available during the study period were selected.

The data collection process took about 7 months from February to August 2017, using various data collection methods, such as focus group discussion (FGD), individual interviews and document analysis. Focus group discussion is a carefully planned series of discussions with 5 to 15 participants designed to obtain their views or experiences of a defined area of interest in a non-threatening environment (Bless, Higson-Smith & Sithole 2013; Polit & Beck 2016). Participants were selected into homogenous groups to promote a comfortable group dynamic and freely share their thoughts about the phenomenon under investigation (Alasuutari, Bickman & Brannen 2009; Polit & Beck 2016). Three FGDs were conducted in this study, and each focus group was comprised of eight participants.

Focus group discussions and individual interviews, which lasted for 45–60 min, and audio was recorded, were guided by the interview guide. Individual interviews and FGDs were conducted with both groups of participants. The individual interviews were conducted at the participant’s preferred venue. The main questions asked were, ‘What is your understanding of a competency-based PHC-oriented nursing education programme?’ ‘What do you consider the features of a competency-based PHC-oriented nursing education programme?’ ‘What is the impact of a competency-based PHC-oriented nursing education programme?’ Probing, paraphrasing, rephrasing and summarising techniques were used during the FGDs to facilitate discussions and generate the needed information. A research assistant took field notes during the discussion to be used as backup information during data collection. The principle of data redundancy guided data collection. The researcher transcribed the audio recordings verbatim to identify response variations and make field notes available for audit checks to ensure validity. Data were subsequently exported to NVivo version 11 for further analysis.

The document analysis included the nursing and midwifery curriculum, students’ learning contracts and study guides. The content of the nursing and midwifery curriculum was analysed following the steps for document analysis (Bowen 2009). Data from the documents leads to probing questions in the individual interviews and FGDs. The sentences and paragraphs from the documents were reviewed, and the primary codes were checked and rechecked in a back-and-forth interplay. Data from the document was scrutinised and compared with data from the individual interview and FGDs to organise them into categories and sub-categories. The categories and sub-categories were compared for similarities and differences. The qualitative data obtained from the FGDs were analysed using qualitative content analysis (QCA). The data were analysed simultaneously with the data collection, allowing the researcher to make adjustments along the way (Maree 2016) premised on the saturation of the theoretical concepts (Strauss, Corbin & CPS 1991). The process of analysing qualitative data involves four primary steps. The first step is decontextualisation, which entails becoming acquainted with the data. The second step is recontextualisation, which means discarding irrelevant information. Categorisation follows swiftly after and encompasses condensing and classifying the data. Finally, compilation consists of writing up your observations in a coherent manner (Bengtsson 2016; Graneheim, Lindgren & Lundman 2017).

### Trustworthiness

Trustworthiness criteria steps, as proposed by Lincoln and Guba (1985), were followed in this study to ensure the quality of the findings. The current study ensured credibility by verifying collected data and emerging findings with the informants and matching it against verbatim transcripts. Having a co-coder also assisted in confirming the emerging codes and categories and a critical reader who drew the researcher’s attention to data that required verification to ensure confirmability of the findings. Having a clearly articulated plan, research methods, data collection and analysis process in the form of a research proposal with the help of experts in qualitative research enhanced the dependability of the study. Transferability was ensured by providing a thick description of the study context, settings, procedures and findings.

### Ethical considerations

Ethical clearance was sought from the University of KwaZulu-Natal Humanities and Social Sciences Research Ethics Committee (reference number: HSS/0064/016D). Permission was obtained from the Registrar and the Academic Head of School. Ethical principles were observed throughout the study. An information sheet was given to participants, and all participants signed consent. Informed consent involved voluntary participation and recording of the interviews. They were informed that they were free to withdraw from the study at any time without recrimination. Privacy and confidentiality were maintained throughout the study by providing anonymous quotes. Data from the study were stored securely in a password-protected file, so no third party could access them. The data will be stored for 5 years according to the university policy before being discarded.

## Results

The students’ ages ranged from 18 years to 23 years, while the educators’ ages ranged from 35 years to 62 years. Thirty-four of the students and educators were females. Most educators have a master’s degree as their highest educational qualification, while their years of experience range from 6 to 31 years.

Two categories emerged from the data: (1) attributes of a competency-based PHC-oriented programme and (2) outcomes of a competency-based PHC-oriented programme. A summary of the categories and sub-categories that emerged is provided in Table 1.

**TABLE 1:** Summary of the emerged categories and sub-categories.

Categories	Sub-categories
Attributes of a competency-based PHC-oriented programme	A tool for PHC re-engineering in South Africa
	Means to revitalise nursing contribution to health service delivery
	A solution to skewed nursing and midwifery workforce distribution
	Contextualised nursing education programme
	Socially responsive curriculum
	Programme with a strong social accountability focus
	Performance-based curriculum
	Student-centred curriculum
	CBC strengthens collaborative partnership
Outcomes of a competency-based PHC-oriented programme	Improved quality of nursing education
	Competent graduates
	Consciousness of the social determinants of health
	Change agents
	Widening access to health care
	Improved quality of care through a collaborative approach
	Strengthened collaborative partnership

CBC, competency-based curriculum; PHC, primary health care.

### Attributes of a competency-based primary health care-oriented programme

The ‘attributes of a competency-based PHC-oriented nursing education programme’ category comprised nine sub-categories. These included: (1) a tool for PHC re-engineering in South Africa, (2) a means to revitalise nursing contribution to health service delivery, (3) a solution to skewed nursing and midwifery workforce distribution, (4) contextualised nursing education programme, (5) socially responsive curriculum, (6) programme with a strong social accountability focus, (7) performance-based curriculum, (8) student-centred curriculum and (9) competency-based curriculum strengthens collaborative partnership.

#### A tool for primary health care re-engineering in South Africa

The CBC was understood to be a tool for PHC re-engineering. This perspective is based on the changes in the health care systems, from a disease-oriented curative focus to a health-oriented and primary health care approach adopted by the country. This led to the nursing department adopting a programme underpinned by primary health care philosophy:

‘With the change to a PHC approach, the re-engineering of PHC agenda … had to revisit our nursing education programme aligning it to the agenda of the health care system …’ (FGD1, P1, Female)

‘[*O*]ur programme is based on PHC re-engineering philosophy in order to produce graduates who can deliver on the health system’s mandate …’ (FGD1, P2, Female)

[*T*]his program emphasises re-engineering of the Primary Health Care in South Africa … through community-based education and community involvement and partnerships. (Curriculum document 2014)

#### Means to revitalise nursing contribution to health service delivery

Data sources revealed that the participants perceived a competency-based, PHC-oriented curriculum as a means to revitalise the contribution of nurses to health service delivery in a climate where society has concerns about the quality of nurses produced and their competency levels in meeting their health needs:

‘[*P*]roduce graduates with a wide range of skills, knowledge and attitudes that will enable them to make a meaningful and sustained contribution to health services.’ (FGD1, P1, Female)

The nurses produced from this new programme are prepared in such a way that they change how nurses are perceived currently that the programmes do not prepare them for the realities in practice. With competencies derived from the needs of the health care system, the graduates produced will definitely make a difference in health service delivery … (Document analysis, 2014)

The programme facilitates the development of transferable life skills required to survive in a challenging health care environment, which has limited resources and complex cases, with the community-based approach, on the other hand, facilitating the development of PHC-related competencies, which are fundamental in current health care system … (Individual Interview, P1, Female)

#### A solution to skewed nursing and midwifery workforce distribution

Data portrayed a competency-based PHC-oriented programme as one of the solutions to the uneven distribution of nurses and midwives, which mainly affects people in remote and rural areas. Unique to the competency-based undergraduate programme was the selection and admission policy that targets students from rural and remote areas as part of opening access to higher education:

‘[*T*]he targeted admission policy in our programme reserves a minimum of 15% of the spaces in the programme for those students from previously disadvantaged communities who meet the entrance criteria to give them a fair chance in competing for spaces. In a way, this policy provides opportunities to address the skewed distribution of nurses and midwives, especially in rural and remote communities where the shortage of nurses is high.’ (FGD1, P6, Male)

#### Contextualised nursing education programme

Data sources reflected the programme as a context-informed programme, as it considered the health care system, the country’s disease profile and the national health priorities. In addition, the graduate competencies used as a building block to the curriculum design were developed from tasks performed by the professional nurse in a PHC-oriented health care system:

‘[*T*]he curriculum considers the health care system of the country… the national health priorities and the quadruple disease burden of the country.’ (FGD3, P2, Female)

‘[*T*]he curriculum prepares the graduates for our country’s context to provide quality care.’ (FGD3, P5, Female)

‘… The graduate of this programme will take full cognisance of the contextual influences on care, such as psycho-social, economic, physical and other factors.’ (FGD3, P4, Male)

#### Socially responsive programme

Other participants perceived a competency-based nursing education programme as a socially responsive programme as it responds to the population’s health needs and equips graduates with skills relevant to respond to the needs of society. According to the participants, the programme uses a range of clinical learning platforms, including community-based learning sites that expose the students to experience and better understand the social determinants of health and their influence on health issues in the communities. They learn through providing services to communities that will address some of the social issues that impact health. According to the data from the interviews, the students learn to work in partnership with communities for sustainability on the implemented projects, with communities taking responsibility for their own health-related issues. According to the interviews, the community-based learning experiences in the programme facilitate the development of students’ abilities as change agents in societies where they exist:

‘The programme produces graduates who are responsive to the health of the. population they live in.’ (FGD1, P3, Female)

‘The students are placed in surrounding communities where they are expected to identify social issues that impact on the health of the communities and come up with a community intervention, with a preventive and health promotive focus.’ (FGD1, P6, Male)

‘The students learn through providing service to under-resourced communities. This helps them better understand what we teach in class as social determinants of health …’ (FGD1, P2, Female)

#### Programme with a strong social accountability focus

The competency-based PHC-oriented programme emerged as a programme with a strong social accountability focus as learning experiences, especially in community-based settings, were aimed at addressing priority health needs and issues in the surrounding community through small-scale community-based interventions by the students. The aim is to develop in students a sense of equity, justice and service ethics as specified in their curriculum:

Students learn through providing service to those with health related issues the students develop that sense of social responsibility. (Document analysis, 2014)

‘The students learn through providing service, which requires profiling the health needs or issues that have an impact on health of their clients at different level.’ (Individual Interview, P1, Female)

The graduates of this programme developed sense of equity, justice and service ethics that will ensure that they work in a responsible and accountable manner. (Curriculum Document, 2014)

#### Performance-based curriculum

The CBC was perceived by some participants as a performance-based curriculum, with success measured by the student’s ability to perform the tasks of a professional nurse presented in the form of competencies in the programme. Central to the curriculum is the ability to perform specific sets of competencies rather than mastery of the content:

This curriculum is about performance, not content assimilated by the students. (Curriculum Document, 2009)

A competency-based curriculum is based on the anticipated roles of the professional nurse in the workplace, which the learners have to master … the desired outcome competencies. (Curriculum Document, 1994)

#### Student-centred curriculum

The programme was considered student-centred in that central to the learning process were the needs of the students, with the students leading and directing their own learning process and acquisition of competencies, with the support and guidance of the facilitators, clinical preceptors and clinical mentors. The programme is characterised by the paradigm shift in the roles, with the students developing their own learning contracts and pacing their own learning. Examples of excerpts from the data are:

‘[*P*]rogramme promotes student-centered learning approach … uses innovative teaching strategies that promote learner autonomy.’ (FGD1, P2, Female)

‘[*W*]e teachers, our role is that of a resource person and a facilitator of learning.’ (FGD1, P7, Female)

‘The students develop learning contracts in line with the learning needs, and they work at their own pace but ensure that at the end of the module, they have covered all the expected competencies.’ (Individual Interview, P2, Female)

#### Competency-based curriculum strengthens collaborative partnership

Implementing a competency-based programme, according to the data sources, strengthened collaborative partnerships between academic institutions, services and surrounding communities, in that stakeholders had input in determining graduate competencies curriculum design and were also involved in facilitating learning in the clinical platforms used for experiential learning:

‘[*W*]e form strong collaborative partnership with our stakeholders… this ensures that our students are well received where we place them for clinical practice.’ (FGD1, P3, Female)

‘The collaborative partnership approach is working well for us. Our partners are supportive of our students because they share a common goal with us.’ (Individual Interview, P1, Female)

### Outcomes of a competency-based primary health care oriented programme

The following seven sub-categories emerged under this category: (1) improved quality of nursing education, (2) competent graduates, (3) consciousness of the social determinants of health, (4) change agents, (5) widening access to health care, (6) improved quality of care through a collaborative approach and (7) strengthened collaborative partnership.

#### Improved quality of nursing education

The document analysis revealed that the implementation of a competency-based PHC-oriented programme improved the quality of nursing education in several ways, including education that focusses on preparing graduates for health service delivery. Implementing a competency-based PHC-oriented curriculum was perceived as a way of correcting the old training characterised by a mismatch between graduate competencies and the health service delivery needs:

… In terms of the learning process the old programme was traditional, using mainly classroom lectures and laboratory demonstrations as teaching methods, with limited clinical supervision to support it. (Curriculum Document, 1994).

… However, the new programme is problem-based, with teaching done in small groups around actual clinical problems. The curriculum focuses strongly on the process of learning, but a reasonable coverage of content is achieved by the purposive clinical allocation of students. (Curriculum Document, 2014)

#### Competent graduates

Data revealed that the competency-based PHC-oriented programme equipped graduates with a range of competencies required from professional nurses in the health care system in the country. Some participants even singled out the issue of language, that the programme structure and learning experiences in diverse settings dealing with clients using different languages assisted them in learning the language used by their clients for effective communication. Here are some of the supporting extracts:

‘… We produce a well-rounded, confident, competent and skilful graduate that embraces the government’s strategy of long and “healthy life for all” in line with PHC re-engineering policy…’ (FGD1, P6, Male)

‘… Our communication in the language of the patient has improved due to the exposure to different learning sites, learning experiences and patients using both English and IsiZulu, demanding us to express ourselves in their language when interacting with them and providing care.’ (FGD2, P1, Female)

#### Consciousness of the social determinants of health

According to the data sources, the programme made students more aware and conscious of the social determinants of health. Through this programme, it emerged that the students learned that each patient or client is unique and requires individualised care that is informed by the surrounding determinants of health. Exposure of students to different communities, especially the under-resourced and disadvantaged communities, raised the students’ consciousness of the importance of understanding the patient’s background for effective care provision instead of using a one-size-fits-all approach:

‘Instead of looking at a patient with TB, we look at the patient presenting with TB because of the environment that he is coming from, then how do we look at the patient holistically?’ (Individual Interview, P2, Female)

‘[*T*]he time that I was exposed to under-resourced communities. It certainly raised my consciousness and helped me to know why we should treat each client as an individual.’ (FGD2, P2, Female)

#### Change agents

The quality of nursing education was also perceived to have improved in that the programme facilitated the development of students as change agents through the community-based approach. The programme inculcated the culture of health promotion and illness prevention, with students working with individuals, families, groups and communities in promoting healthy living and lifestyles. According to the data from the interviews, the students in community-based settings provided health promotion talks and free health screening, with some cases referred to health facilities for further management:

‘ … Learning experiences develop the culture of students as change agents that will advocate for health.’ (Individual Interview, P2, Female)

‘… [*H*]ealth promotion has become part of us now when we are nursing a patient, we do it unconsciously most of the time … it has become our second nature’ (FGD3, P1, Female)

‘During the breastfeeding week, as UKZN students, we gave mothers talks every morning on the importance of breastfeeding, in postnatal care units, we assisted mothers with newborn babies with initiating breastfeeding as most of the young seemed to struggle putting babies on the breast, we educated and assisted others who had cracked nipples.’ (FGD3, P4, Male)

‘[*S*]tudents like giving health education. It is how we were taught from first year. We believe in health promotion and illness prevention.’ (FGD3, P4, Male)

#### Widening access to health care

Learning through providing service in under-resourced community-based settings was viewed as widening access to those with limited access to care. The final-year students narrated their 2 weeks’ experience in the Primary Health Care Phelopepa train, which moves around the country, providing health services to rural areas. According to the participants, this train has a team of health professionals, comprising senior students from various health sciences programmes from different institutions in the country, learning by providing services under the guidance of professionals to people from rural areas. Through this exposure, the student nurses learn to work in multidisciplinary teams and better understand the need for widening access to health care to those in remote and rural areas, using the available limited resources. In addition, it strengthened their leadership abilities, as stated by one of the participants:

‘… Were very fortunate to go to Phelophepa health training, which serves people from rural area with no access to health care … all of us were allocated to own consultations rooms … This experience also made me better understand the leadership role of a nurse in a health team.’ (FGD3, P3, Female)

#### Improved quality of care through a collaborative approach

The participants indicated that the programme produced graduates with the potential to improve the quality of care through a collaborative approach. This approach to care was facilitated through a problem-based approach, which required students to analyse the presenting situation holistically and propose care that will contribute to holistic management of the issue at hand through a multidisciplinary approach:

‘The problem-based learning approach strengthened our collaborative approach to care So, as a nurse a client, you get to collaborate with other people in a multidisciplinary team members.’ (Individual Interview, P1, Female)

‘We use more of case study approach or a problem-based approach, cases, or scenarios. This approach to teaching prepares graduates in a way that will improve care provided to patients’ (Individual Interview, P2, Female)

#### Strengthened collaborative partnership

Implementing a competency-based programme, according to the data from the interviews, strengthened collaborative partnerships between academic institutions and clinical facilities. This partnership improved the reception of the students in the clinical settings, including the quality of support they receive. Participants viewed the collaborative approach as having a ripple effect in that students learn to embrace collaborative partnerships in the care provision:

‘[*W*]e form strong collaborative partnership with our stakeholders…this ensure that our students are well received where we place them for clinical practice.’ (FGD1, P3, Female)

‘The collaborative partnership approach is working well for us. Our partners are supportive of our students because they share a common goal with us.’ (Individual Interview, P1, Female)

## Discussion

Data sources revealed that CBC is understood as a tool for PHC re-engineering because the primary health care philosophy underpins CBC programmes. This increases the production of graduates who are responsive to the health care needs of the population (Saloojee 2020; Rispel 2015). Such graduates can conduct health promotion and illness prevention for clients and communities. The CBC was further conceptualised as a solution to addressing the skewed distribution of nurses and midwives. This is because students are strategically recruited from rural and remote areas with a high shortage of nurses (Caccia et al. 2015; Muraraneza & Mtshali 2018a). In CBC, students are provided with academic monitoring support and guidance. This increases the production of graduates who are fit for practice and thus reduces nurse shortages in under-resourced communities to strengthen the health system (Bvumbwe & Mtshali 2018; Frenk et al. 2010).

The CBC is understood as a context-based curriculum in that it considers the contextual features of the setting where learning takes place. With CBC, education is affected by society’s expectations. This increases the production of competent nurses to work in under-resourced communities with limited access to health care. Findings from this study showed that CBC is conceptualised as needs-driven as students’ needs are considered. This allows students to be actively involved in their own learning, which results in the production of students who are equipped with transferable life skills needed in today’s workplace (Muraraneza & Mtshali 2018b). This study further reported that CBC is considered performance-based because performance is deemed successful when students can demonstrate what they have learned in a measurable performance of discreet skills because with CBC, success is measured by the student’s ability to perform the tasks as a professional nurse and not to regurgitate the content (Gravina 2017; Muraraneza & Mtshali 2018b).

Data sources reported that CBC is student-centred learning (Stanley & Dougherty 2010) as it allows students to take a central role in teaching and learning. With CBC, students construct their own knowledge as the teacher takes a more facilitative role. Furthermore, CBC uses innovative teaching strategies such as Competency based learning (CBL), Problem based learning (PBL) and CBE, which facilitate deeper learning and learner autonomy, resulting in the production of graduates committed to lifelong learning (Baloyi & Mtshali 2018; Caccia et al. 2015b; Mtshali & Gwele 2015). Findings from this study showed that adopting CBC eliminates concerns about approaches promoting hospital-focussed care that pay less attention to the social determinants of health. This is congruent with another study which states that with CBC, the focus is more on the improvement of the quality of nursing education (Gervais 2016), as it uses learner-centred pedagogies, which promote intelligent engagement and thus prepare graduates who will meet the health needs of the country.

The data sources in this study highlighted that nursing education using CBC approaches would be harmonised with national health policies, which called for nursing education transformation and preparation of nursing graduates. Findings highlighted that programmes aligned to national health priorities result in the production of competent graduates who are conscious of social issues that affect the health of the communities and come up with necessary interventions. In addition, the findings of this study revealed that such graduates become change agents, which is consistent with the studies of Frenk et al. (2010) and World Health Organization (2009, 2010), which also agree that CBE produces students who are change agents within these communities as they identify social injustices within the communities and be able to challenge these to implement changes (Boelen 2016). According to data sources, CBC in the undergraduate nursing programme is regarded as a vehicle aimed at producing 21st-century nursing graduates who are competent with knowledge, skills and attitudes for the current and future health care system. Findings further highlighted that CBC strengthens collaborative partnership, which produces graduates who are highly skilled to collaborate with individuals and communities in promoting the country’s long and ‘healthy life for all’ agenda. This resonates with the findings of Barton et al. (2018) and Tan et al. (2018), where CBC was highlighted to produce competent and collaborative nurses.

### Limitations

Factors such as the availability and accessibility of resources, including human (research participants) and technological resources limit this study’s findings. These limitations were because of the small number of institutions and participants used in the current study. The findings of this study are not very recent and therefore should be used with caution. Furthermore, only a few respondents were included in the study, which prevented a wide sampling of opinions from educators with diverse backgrounds, as only one institution was used.

Therefore, to address these limitations, further research should be conducted with broader stakeholders, including academics and practitioners from other higher education institutions. The study should also consider larger-scale surveys to explore views nationally on competency-based PHC programme designs in South Africa. Additionally, research can be carried out to see if or how best changes or amendments can be made within the existing competency-based PHC nursing course structures to make them more satisfactory for nursing students, employers and society.

## Conclusion

This article explored the students’ and educators’ perspectives on competency-based PHC-oriented nursing education programmes in the South African context. The stakeholders portrayed the competency-based PHC-oriented curriculum as a tool for PHC re-engineering in South Africa. This perspective is based on the changes in the health care systems, from a disease-oriented curative focus to a health-oriented and primary health care approach adopted by the country, which led to the nursing department adopting a programme underpinned by primary health care philosophy. From the findings of the study, CBE was seen as a tool for PHC re-engineering, a solution to skewed nursing and midwifery workforce distribution and a performance-based curriculum with the capability of producing competent graduates who will act as change agents to improve the quality of nursing care. A competency-based nursing education programme is considered a socially responsive programme in that it responds to the population’s health needs and equips graduates with a set of skills relevant to respond to the needs of society. If implemented, the findings of this study will assist in strengthening the PHC re-engineering, which will improve access and quality of care provided to the population.
